# Conceptual Model of Hearing Health Inequalities (HHI Model): A Critical Interpretive Synthesis

**DOI:** 10.1177/23312165211002963

**Published:** 2021-05-28

**Authors:** Dialechti Tsimpida, Evangelos Kontopantelis, Darren M. Ashcroft, Maria Panagioti

**Affiliations:** 1 Centre for Primary Care and Health Services Research, Institute for Health Policy and Organisation (IHPO), School of Health Sciences, Faculty of Biology, Medicine and Health, The University of Manchester, Manchester, United Kingdom; 2 Institute for Health Policy and Organisation (IHPO), School of Health Sciences, Faculty of Biology, Medicine and Health, The University of Manchester, Manchester, United Kingdom; 3 NIHR Greater Manchester Patient Safety Translational Research Centre, School of Health Sciences, Faculty of Biology, Medicine and Health, The University of Manchester, Manchester, United Kingdom

**Keywords:** critical interpretive synthesis, health inequalities, healthy aging, health literacy, patient safety

## Abstract

Hearing loss is a major health challenge that can have severe physical, social, cognitive, economic, and emotional consequences on people’s quality of life. Currently, the modifiable factors linked to socioeconomic inequalities in hearing health are poorly understood. Therefore, an online database search (PubMed, Scopus, and Psych) was conducted to identify literature that relates hearing loss to health inequalities as a determinant or health outcome. A total of 53 studies were selected to thematically summarize the existing literature, using a critical interpretive synthesis method, where the subjectivity of the researcher is intimately involved in providing new insights with explanatory power. The evidence provided by the literature can be summarized under four key themes: (a) There might be a vicious cycle between hearing loss and socioeconomic inequalities and lifestyle factors, (b) socioeconomic position may interact with less healthy lifestyles, which are harmful to hearing ability, (c) increasing health literacy could improve the diagnosis and prognosis of hearing loss and prevent the adverse consequences of hearing loss on people’s health, and (d) people with hearing loss might be vulnerable to receiving low-quality and less safe health care. This study uses elements from theoretical models of health inequalities to formulate a highly interpretive conceptual model for examining hearing health inequalities. This model depicts the specific mechanisms of hearing health and their evolution over time. There are many modifiable determinants of hearing loss, in several stages across an individual’s life span; tackling socioeconomic inequalities throughout the life-course could improve the population’s health, maximizing the opportunity for healthy aging.

Hearing loss involves the partial or total inability to hear sounds from one or both ears. It can be categorized as mild, moderate, severe, or profound, according to its severity. Approximately 15% of the global adult population suffers from some degree of hearing loss (World Health Organization [WHO], 2013). Approximately 432 million adults—almost 7% of the global population—has disabling hearing loss, defined as a pure-tone average (PTA of the audiometric hearing threshold at 500, 1000, 2000, and 4000 Hz (PTA 0.5–4.0 kHz)) greater than 40 dB HL in the better hearing ear (Wilson et al., 2017).

Hearing loss is far beyond a sensory disorder, as it is associated with negative physical, social, cognitive, economic, and emotional consequences. In high-income countries, hearing loss is the third most common chronic health condition among older adults, following high blood pressure and arthritis (Barnett et al., 2014). Nevertheless, it should be noted that the magnitude of the effect of age on hearing loss varies considerably among individuals. Nearly one in three people older than 70 years old do not develop high-frequency hearing loss, a condition that has traditionally been linked with aging (Slade et al., 2020). Despite diligent research over the past decades, *our understanding of age-related hearing loss is very limited* (Bowl & Dawson, 2019, p. 1).

What is widely known as *age-related hearing loss* has similar characteristics to sensorineural hearing loss that can occur at any age. Based on that, as the knowledge on the causes of hearing loss in patients with older age increases, the need for expressions of hearing problems without specific etiology on older age, through terms such as “presbyacusis,” is likely to be diminished (Kiessling et al., 2003). It might be helpful to consider the injurious influences in hearing during individuals’ life spans. The earlier notion, though, has a long history in hearing research, when the concept of “socioacusis” first introduced to define “the hearing loss that develops over time after repeated exposures to loud noise and not to occupational exposure to noise, physiological changes with age, or disease” (Abbate et al., 2005). Rosen and Olin’s (1965) studies in the 1960s revealed that the members of the Mabaan tribe in southeast Sudan, living in a dramatically quiet atmosphere, had a significantly superior hearing at 70 years old compared with people with a similar age who lived in noisy industrial areas.

More recently, the term of *lifestyle-related hearing loss* has been added to the literature, where lifestyle refers to *social practices and ways of living adopted by individuals that reflect personal, group and socioeconomic identities* (Tsimpida et al., 2019b). The term incorporated the notion of *socioacusis* by including the hearing loss cases that develop due to exposures to sociospatial and modifiable lifestyle factors (Tsimpida et al., 2020b). In practice, many actions could be taken on several levels to make social listening safe, in terms of intensity, duration, and frequency of exposure to sounds (WHO, 2015b). The WHO has suggested that primary prevention could reduce hearing loss prevalence by 50% or more in some world regions (Wilson et al., 2017).

On the other hand, evidence shows that a considerable percentage of hearing loss cases that cannot be prevented can be managed satisfactorily with hearing aids, which is vital given the substantial burden hearing loss causes (Wilson et al., 2017). However, because the cost of rehabilitative services for hearing loss is high, all countries, especially the resource-constrained countries, should focus on primary prevention rather than tertiary prevention (WHO, 2018). The focus on preventing major causes of deafness and hearing loss reaffirms WHO’s vision of a world in which *no person experiences hearing loss due to preventable causes* (WHO, 2018). There is great potential for reducing the burden of hearing loss; in order to do so, modifiable factors linked to socioeconomic inequalities in hearing health need to be better understood and addressed (Emmett & Francis, 2015; Scholes et al., 2018). 

Many researchers have tried to identify the mechanisms that link early-life experiences to health in older age. Various conceptual models on life-course epidemiology have been formulated (Ben-Shlomo & Kuh, 2002). These models aim to facilitate a different understanding of the causal mechanisms. The life-course approach to chronic disease epidemiology examines an individual’s life history by investigating how early-life events and social determinants of health influence their future decisions and health issues such as diseases. This approach suggests that the diseases that appear in an individual’s adult life may originate from their early-life experiences (Kuh & Ben-Shlomo, 2004).

The *theory of causation* (Figure 1) is another prominent theoretical framework that explains health inequalities. It proposes that social stratification formulates a social gradient in health, having a primary cause of the unequal distribution of power, money, and resources (Kröger et al., 2015). Another significant model is Diderichsen’s model of *the mechanisms of health inequality* (Diderichsen et al., 2001; Diderichsen & Hallqvist, 1998), which explains the several mechanisms that play a role in stratifying health outcomes. Diderichsen’s theory describes how the political context contributes to health inequalities (WHO, 2010).

**Figure 1.**
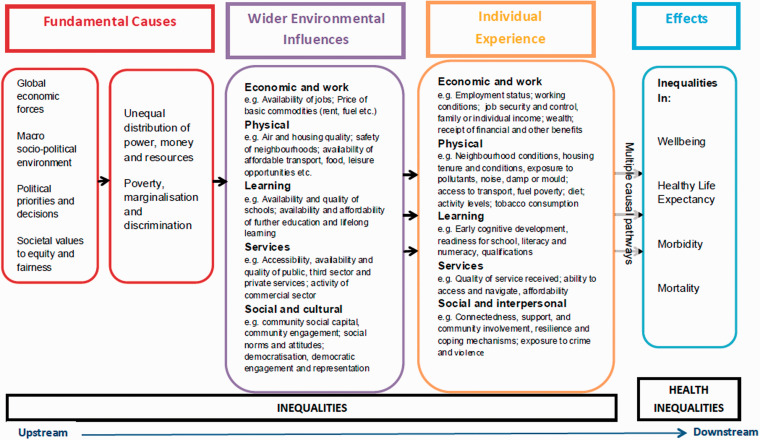
Health Inequalities: Theory of Causation (Molony & Duncan, 2016).

Recent models on health inequalities focus on the *individual’s* perspective, that is, on one’s education, employment, and income (Diderichsen et al., 2012). This perspective emphasizes the relationship between one’s social position and health, as showcased by Åberg’s model (Åberg Yngwe, 2004; Figure 2). However, Åberg’s model does not explain the evolution of the early-life socioeconomic circumstances, in terms of the disadvantages of material aspects over time, which is being highlighted as a crucial issue in life-course literature (Cheval et al., 2019).

**Figure 2.**
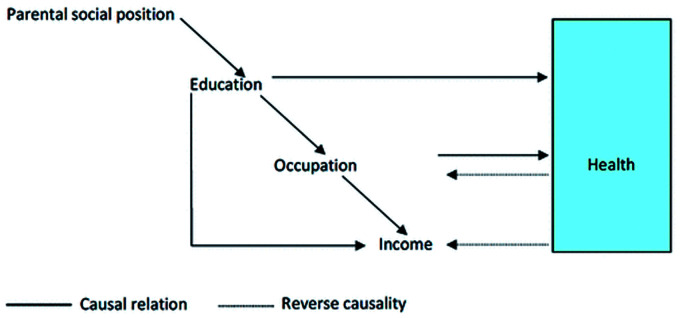
Social Position and Health and Relevant Causal Mechanisms (Åberg Yngwe, 2004).

## Purpose of the Present Study 

Today, after decades of research, the burden of adult-onset hearing loss is high, and the etiology of what is widely known as “age-related hearing loss” remains unclear (Olusanya et al., 2014). The level of uncertainty regarding potential mechanisms has led to the need to conduct a critical analysis of the existing literature (Dixon-Woods et al., 2006). The goal is to assign explanations of the impact of the early-life socioeconomic circumstances on one’s hearing health, and how hearing health inequalities are perpetuated throughout one’s life-course.

A conceptual model’s importance emerged due to the multifaceted factors that contribute to hearing health disparities (Diez Roux, 2012; Dixon-Woods et al., 2006). A conceptual model can provide a visual representation of the multiple factors that affect a person’s hearing in different life stages. Furthermore, a conceptual model offers the framework to generate testable hypotheses and empirically valuable questions to inform future research, interpret results, and design targeted interventions (Diez Roux, 2012).

The nature of hearing loss also reinforces the need for a separate model for hearing health inequalities; it is a noncommunicable disease (WHO, 2015a) with long duration and slow progression during the life-course and can seriously affect one’s lifestyle. The individuals who have hearing loss are more likely to have poorer educational achievements, higher unemployment rates, and lower annual family income than those without hearing loss (Bartley & Blane, 2008). Moreover, there is a considerably higher prevalence of multimorbidity among adults aged 65 and older who suffer from hearing loss, compared with those who do not suffer from hearing loss or with other health conditions, which increases the overall disease burden (McKee et al., 2018; Young, 2014).

Therefore, hearing health inequalities cannot be satisfactorily contextualized within more general models on inequality (Diez Roux, 2012). A new analytical approach, which embraces the notion of structural causation and articulates the mediating mechanisms of the cumulative hearing inequalities and their evolution over time, is needed (Diez Roux, 2012). Given the burden of adult-onset hearing loss, such a conceptual model for identifying hearing health inequalities could improve many indicators of population health status, including the broad measures of individual’s physical, mental, and social well-being.

This review aims to (a) provide an interpretive synthesis of the existing literature and give insight into the socioeconomic disparities in hearing health and (b) formulate a conceptual model for hearing health inequalities, which depicts the specific mechanisms for hearing health and their evolution over time.

## Research Design and Methods

This review’s scope is broader than testing a specific research question, which could be achieved through a systematic review or scoping review. This article aims to integrate diverse forms of research evidence. To do so, the methodology of critical interpretive synthesis (CIS) is adopted (Depraetere et al., 2020; Flemming, 2010). The CIS is a relatively new review type used for synthesizing multimethod research that has its origins in health equity research and is increasingly applied in the social sciences (Depraetere et al., 2020). This review type is distinguished from other review types through its emphasis on theory development and flexibility, involving an iterative approach to searching and selecting evidence (Depraetere et al., 2020).

This method uses theoretical sampling and appraises the quality of evidence based on its relevance to the investigation topic. The quality of research is appraised as the extent to which it informs theory and involves the development of *synthetic constructs* or *themes*. These themes are then linked and supported by the relevant evidence, which is placed within its context to build a highly interpretive conceptual model (Dixon-Woods et al., 2006). The authors developed this approach and rejected the concept of a reciprocal translational analysis, that is, a summary of what has been already used in the literature because the latter is not helpful when dealing with a diverse body of evidence and attempting to develop a theory (Dixon-Woods et al., 2006). The CIS, instead, is *explicitly oriented towards theory generation* and adopts a methodology with some steps similar to those of a systematic review in combination with qualitative interpretive approaches, aiming to review and combine existing evidence into a coherent whole, and to provide new insights with explanatory power (Barnett-Page & Thomas, 2009).

This article relies on CIS reviews’ described guidelines to ensure that the reporting is transparent and coherent (Depraetere et al., 2020). The following six activities represent the dynamic process of a CIS:

1. *Open research question*: The CIS starts with the formulation of an open research question regarding the impact of socioeconomic inequality on hearing loss.
2. *Literature search*: We searched three databases—PubMed, Scopus, and Psych—using the keywords *hearing *AND* inequalities,* *hearing* AND* disparities,* and *hearing* AND *determinants* in the title/abstract. We identified 779 articles with potentially relevant abstracts. The most recent search was conducted in October 2020.
3. *Literature selection*: The literature was selected following the inclusion criteria given later, not necessarily aiming to identify and include all relevant literature, but rather sources directly relevant to the theoretical framework. We included peer-reviewed studies (empirical studies, systematic reviews, and theory-based overviews), which included participants with hearing loss (with no age restrictions) and presented associations between hearing loss and health inequalities either as a determinant factor or as a health outcome. English-written articles from any country and setting were eligible for inclusion in this study. A two-stage screening process was applied: First, titles and abstracts were screened against the inclusion criteria; second, a detailed review of the potentially eligible full-texts was completed. Two reviewers were involved in the data screening process (D. T.; M. P.). Disagreements were resolved through discussion until a consensus was reached. If the two authors could not reach a consensus, the team of four coauthors would discuss until a consensus was reached.
4. *Quality appraisal*: We assessed the methodological quality of the included studies, using criteria provided in the guidance on quality assessment components and ratings (Thomas et al., 2004). The studies were assigned a rating of 1 for each one of the following main criteria met (maximum rating of 4):
  - *Selection bias*: likely to be representative of the target population and have a response rate or data capture among eligible participants of 70% or greater;
  - *Design*: cohort analytic, case-control, cohort, or an interrupted time series;
  - *Covariates*: control of a minimum of three critical covariates in the analysis, including sociodemographic characteristics (e.g., age, sex, and education);
  - *Data collection methods*: use of psychoacoustic hearing assessment tools, which are valid and reliable.

The aforementioned quality criteria do not examine the theoretical contribution to CIS, thus were not used to exclude studies.

5. *Data extraction*: A data extraction table was developed, including the following elements of the selected studies: names of authors, publication year, country, and key point(s) made by the authors and in which synthetic constructs they were applied. Four recurring concepts/themes were identified from the studies, and the literature was placed within its context, to inform the emerging research themes (Dixon-Woods et al., 2006). Supplementary Table 1 provides the key discussion points for the analysis of the 53 studies, which support the four research themes. Supplementary Table 2 presents the scoring criteria of quality appraisal.
6. *Formulation of a synthesizing argument*: The separate analysis of the sources used in each theme helped to identify the relationships between the four themes. A synthesizing argument was then formulated, which also takes into consideration elements of theories on health inequalities. The themes were then synthesized in an inductive approach, and a coherent theoretical conceptual model was formulated, depicting the relationship between the network of the discussed constructs, which aims to contribute to the theoretical development of the synthesis topic (Depraetere et al., 2020).

### Definition of Key Terms

The term *socioeconomic position (SEP)* is used instead of the term *socioeconomic status*, to refer specifically to the components of economic and social well-being; this is in line with the suggestion of Krieger et al. (1997). The term *SEP* is linked to both childhood and adult social class positions. It includes both resource-based (e.g., deprivation) and prestige-related characteristics, which refer to the individual’s rank or status in a social hierarchy. We decided to include education, occupation, income, and wealth as the selected indicators of SEP; according to the list of SEP indicators proposed by Galobardes et al. (2006), these factors encompass aspects of an individual’s socioeconomic stratification throughout their life-course.

We use the term *hearing loss* instead of the term *hearing impairment*, which looks beyond pathology, addressing issues that interact to affect the individual’s ability to maintain as high a level of health and well-being as possible and function within society: According to the Sociopolitical Model of Disability, hearing disability is being approached through the lens of the *loss or limitation of opportunities,* rooted in societal barriers (Smart, 2006). We consider this approach more suitable given the aims of this study, which are to examine the social determinants of hearing health. This approach is also consistent with the International Classification of Functioning, Disability and Health and Core Sets for Hearing Loss, which highlight the importance of a multidimensional model for assessing the functioning and disabilities of people with hearing loss (Alfakir et al., 2019; Granberg et al., 2014).

## Results

**Figure 3.**
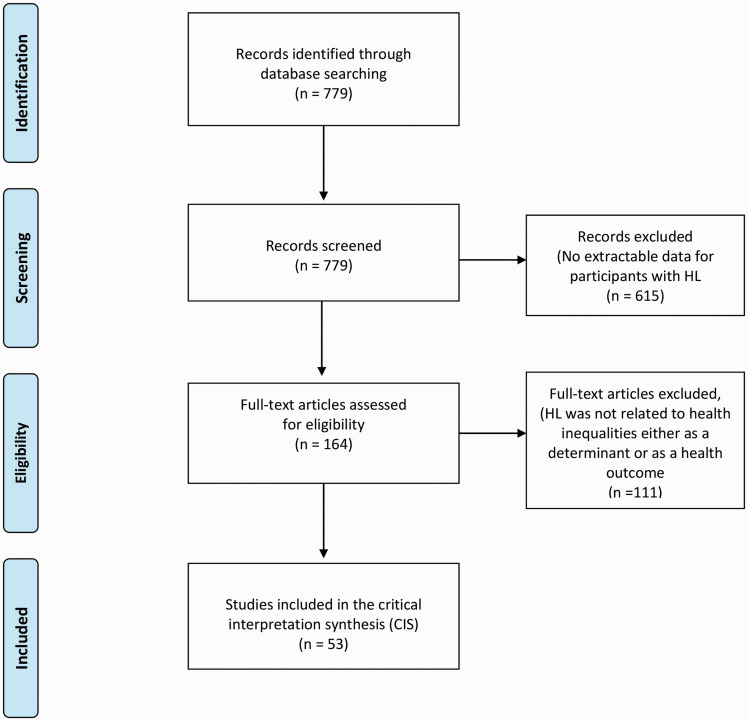
Study Identification Flow Diagram, Inclusion and Exclusion Criteria. HL = hearing loss; CIS = critical interpretive synthesis.

A total of 779 studies were identified, and following the two-stage screening process, 53 studies were selected for inclusion in the review, which coincides well with the ideal number of around 50 studies that should be included in a CIS (Dixon-Woods et al., 2006). Figure 3 shows the flow diagram of the study identification and selection process. The findings from the CIS are provided later in the form of synthesizing arguments, which are based on the following four themes, which are then linked and used for the development of a conceptual model (Depraetere et al., 2020):

### Theme 1 (T1): Low SEP and Hearing Loss Form a Vicious Cycle, as Hearing Loss May Be Both a Consequence of and a Causal Contributor to Socioeconomic Disparity

Prior research emphasizes the health disparities that exist between people with and without disabilities (Dobbertin et al., 2015). The need for attention to disparities within a population with a disability has been underestimated, and there is a lack of research on the disparities related to the type of disability (Horner-Johnson et al., 2013). A number of researchers have reported that low SEP is associated with increased risk of inequalities in the hearing health of midaged people (Chou et al., 2015; Kupriianova et al., 2013; Scholes et al., 2018).

More specifically, some critical indicators of the socioeconomic stratification (Galobardes et al., 2006), such as education, occupation, income, and wealth, have been correlated to hearing loss; for instance, people with less access to education have relatively worse hearing health (Andrade & Lopez-Ortega, 2017; Cruickshanks et al., 1998; Scholes et al., 2018; Tsimpida et al., 2019b). Furthermore, people who have attained a higher level of educational are less likely to suffer from hearing loss in their adult lives (Chou et al., 2015; Martin et al., 2012; Zhan et al., 2011).

Lower educational attainment is a predictor of social inequality in later life, as it affects employment opportunities and earning potential, limiting people with lower education to less paid jobs. Moreover, a lower level of education is associated with occupations that involve high noise exposure levels, thus increasing an individual’s risk of acquiring hearing difficulties (Pierre et al., 2012). High exposure to noise may explain why people with less education suffer more from hearing loss (Martin et al., 2012), as there is a clear relationship between occupational exposure to noise and an increased likelihood of suffering from hearing loss (Cruickshanks et al., 1998; Helvik et al., 2009; Hétu et al., 1988). Notably, in Rosenhall et al.’s (1999) study, the manual workers had a similar level of self-assessed hearing difficulties as nonmanual employees 10 years older. What is currently unknown is whether the hearing level of industrial workers exposed to hearing health promotion interventions and wear protective equipment differs from workers or the general public who have not undergone such interventions.

Occupation is closely related to income and wealth, which are important determinants of populations’ average health and contribute significantly to health inequalities (Wilkinson & Pickett, 2006). Existing literature shows that the financial constraints and inadequate health insurance may affect individuals’ willingness to seek help for hearing loss (Chan et al., 2017) and lead to lower hearing aid acquisition and usage (Bainbridge & Ramachandran, 2014). This might explain the higher prevalence of untreated hearing loss among low-income adults, compared with those in the highest income and wealth quintiles (Nieman et al., 2016; Scholes et al., 2018; Tsimpida et al., 2019b). The consequences of untreated hearing loss vary and depend on the degree, type, and configuration of loss. However, hearing loss may significantly affect the ability of individuals to maintain good health and to function within society, as it limits their ability to participate in interpersonal relations, and diminishes their health-related quality of life (Danermark et al., 2013; Eisele et al., 2015; Tsimpida et al., 2018b). This phenomenon can be explained within a broader bio-psycho-social-environmental context, consistent with the WHO’s definitions of disability (International Classification of Diseases, 11th Revision, 2018).

Hearing loss is associated with significant adverse outcomes. For example, hearing loss in early life may lead to low educational achievements (Chou et al., 2015; McKee et al., 2018; Pierre et al., 2012; Smith et al., 2016) and may affect an individual’s future employment opportunities and even their ability to continue working or to advance occupationally (Emmett & Francis, 2015; McKee et al., 2018). People who have hearing loss often use a ream of strategies to live and work with it, facing numerous challenges in order to maintain optimal work performance (Shaw et al., 2013). These challenges may affect people’s decision to retire early, subsequently affecting their financial position as older adults (Davis et al., 2016; Emmett & Francis, 2015; McKee et al., 2018; Smith et al., 2016). On the other hand, people with good hearing may have better chances to achieve higher status positions (Chou et al., 2015). Thus, in line with the *health selection* approach in health inequalities, differences in SEP might result from a lower hearing health status, which suggests that differences in health affect the SEP (Kröger et al., 2015).

Hearing loss, especially when left unaddressed, may limit one’s ability to communicate, making things worse for those who have hearing loss and other chronic health conditions commonly comorbid with hearing loss. This may even delay their detection. The delay in detecting health issues could also lead to further socioeconomic disparities in patients with hearing loss, by increasing the disease burden and lowering their health-related quality of life (McKee et al., 2018; Tsimpida et al., 2018b; Young, 2014). Therefore, the already significant burden of having a chronic disease for the more socially and economically disadvantaged could worsen, contributing to enhancing inequalities in morbidity and mortality (Beauchamp et al., 2015). It could be argued that SEP and hearing loss form a vicious cycle, with each causing the other: Hearing loss is *both a consequence and a causal contributor to socioeconomic disparity.* Besides, not only can a sensory impairment lead to low economic resources in adulthood (Chou et al., 2015) but also the hearing health inequalities can be accumulated: The more a person functions in a lower SEP during their life span, the more their hearing problems will be accumulated.

### Theme 2 (T2): Indicators of Lower SEP Are Associated With a Less Healthy Lifestyle, Which Is Harmful to Hearing Ability

The associations between indicators of lower SEP and hearing loss may indicate exposure to risk factors that have a damaging effect on hearing (e.g., exposure to loud noise during the employment in noisy occupations; Lie et al., 2016). However, they may also indicate less healthy lifestyle factors, which are the nonmedical determinants of health (Tsimpida et al., 2018b). Evidence shows that several modifiable lifestyle factors—such as smoking (Gopinath et al., 2010), alcohol consumption (Zhan et al., 2011), having a high body mass index (BMI), eating high fat and high-calorie food (Curhan et al., 2013; Üçler et al., 2016), and insufficient exercise (Curhan et al., 2013; Spankovich & Le Prell, 2013)—increase the likelihood that a person will have poor hearing health. Hence, adopting a healthy lifestyle, not smoking, maintaining proper nutrition, and exercising regularly, can minimize the lifestyle risk factors for hearing loss in older adults (Davis et al., 2016). Existing studies have investigated the cross-sectional relationship between higher physical activity and hearing sensitivity and suggest that hearing accessibility to fitness programmes may not enable people with sensory losses to participate effectively.

Moreover, the impact of alcohol consumption on hearing thresholds in older age is not yet clear. Studies that have examined this association are generally of poor quality and do not allow for satisfactory analyses and result in controversial findings. For example, the drinking measure used in Ecob et al.’s (2008) study was the number of standard units of alcohol consumed in a typical day at the age of 45 years, coded to “greater than or equal to seven drinks per day” in contrast to “all other.” Another study that examines a cohort of the European population was also poorly designed and concluded that moderate alcohol consumption—defined as “at least one alcoholic drink a week”—was seen to have a protective effect on hearing (Fransen et al., 2008). Also, even though Tsimpida et al.’s (2019b) recent study shows that drinking above the low-risk-level guidelines—that is, more than 14 units of alcohol in the last 7 days—increases the likelihood of hearing loss, the cross-sectional nature of the study does not allow for the generalization of the findings. By contrast, the longitudinal study of Gopinath et al. (2010) does not confirm the association between alcohol consumption and prevalent hearing loss. It can thus be suggested that, to date, the impact of alcohol intake on hearing loss is not fully understood. Therefore, future large population-based studies are warranted.

People in lower SEP might face conditions that drive them to adopt health-damaging behaviors and avoid the health-protecting ones (Adler & Stewart, 2007). For instance, it may be the case that the lower a person’s income, the less they can afford to buy healthy food, which is almost always more expensive. A lower level of education and income may also lessen one’s engagement in healthy daily behaviors such as physical activity (Zhan et al., 2011). Besides, high levels of stress due to lower resources can induce unhealthy behaviors, such as sugar consumption (Spankovich & Le Prell, 2013) and reliance on tobacco and alcohol (Gopinath et al., 2010), as attempts of short-term stress release. Also, evidence shows that those in a lower SEP, in terms of having a lower level of education and lower income, are more likely to smoke. This phenomenon is not related to the likelihood of smoking initiation, but to the likelihood of quitting, which has been closely related to higher education and higher income levels (Adler & Newman, 2002). The adoption of these behaviors is not due to a lack of willpower or moral fortitude but to a lack of educational opportunities that shape an individual’s earning potential and tend to lead to a lower income (Adler & Stewart, 2007). In general, the lack of material resources, for people who may face pressing problems with income, employment, or even personal safety, lowers their possibility to prioritize and contribute their time and energy to adopting healthy behaviors (Adler & Stewart, 2007).

A recent study finds that socioeconomic and lifestyle risk factors, such as BMI, physical activity, and tobacco and alcohol consumption, are associated with hearing loss among older adults as strongly as core demographic risk factors, such as age and gender. The study argues that lifestyle factors (such as high BMI, physical inactivity, tobacco consumption, and alcohol intake above the low-risk-level guidelines) may account for the higher prevalence of hearing loss among males (Tsimpida et al., 2019b). Moreover, socioeconomic and lifestyle factors may interact. Another study shows in fact that smoking behavior amplifies the damaging effect of occupational noise exposure on hearing (Sung et al., 2013). It can therefore be proposed that lifestyle behaviors act as causal pathways that mediate the relationship between social determinants and hearing health and help to explain the association between them.

### Theme 3 (T3): Improving Health Literacy Can Mitigate Hearing Health Inequalities and Play a Significant Role in the Adoption of Beneficial Hearing Health Behaviors, Including Help-Seeking for Hearing Problems, Hearing Aid Acquisition, and Usage

An increasing number of studies attest the fact that people who are less likely to adopt beneficial health behaviors have low health literacy. The concept of health literacy refers to one’s ability to make judgments and decisions concerning health care, disease prevention, and health promotion in their everyday lives (Van den Broucke, 2014). Previous studies show differences in health literacy patterns within population subgroups, with the most vulnerable demographic groups having lower health literacy (Beauchamp et al., 2015). Therefore, health literacy plays an essential role in explaining the underlying mechanism that drives the relationship between one’s low level of education and poor general, physical, and mental health (Beauchamp et al., 2015; van der Heide et al., 2013). Consequently, health literacy skills may act as modifiers between people’s educational level and their adopted health behaviors (Arcaya et al., 2015).

Health literacy is a multidimensional construct that also refers to one’s ability to navigate the healthcare system and work out the best care for them and their ability to decide which providers they need to see (Beauchamp et al., 2015). For this reason, an individual with limited financial resources may not feel the urgency to seek medical care for a health need. In contrast, the same individual with ample financial resources may feel able to prioritize their health needs (Barnett et al., 2014). Therefore, having a low SEP may not only be a barrier to accessing hearing health care due to financial costs (Barnett et al., 2014), but it may also reflect disparities in people’s access to identification and treatment of hearing problems (Chan et al., 2017; Harrison et al., 2020; Luo et al., 2020). The latter is discussed in Benova et al.’s (2015) study, which examines four SEP indicators—education, occupation, income, wealth—in the health-seeking process of older adults with hearing loss. They find that there is a strong association between SEP and self-report of hearing difficulty for a referral to secondary healthcare services. Thus, people with low SEP are less likely to seek help or access hearing health services (Tsimpida et al., 2019a).

Moreover, after the onset of hearing loss, individuals may face substantial disparities in accessing and using hearing health care (Nieman & Lin, 2017; Tsimpida et al., 2019a). As a result, a person with hearing loss coming from a lower SEP is more likely to experience unmet healthcare needs due to a combination of factors, including income, education, access to health services, and disability. Thus, the disadvantaged social situation of people with functional limitations such as hearing loss is a significant additional barrier to their already limited access to health care (Bainbridge & Ramachandran, 2014; Chien & Lin, 2012; Nieman & Lin, 2017; Nieman et al., 2016; Pichetti et al., 2016). It should be noted that the impact of SEP on hearing aid uptake is closely related to the hearing aid dispensing arrangements in each country. For example, financial constraints and lack of or inadequate insurance coverage are significant barriers to hearing health care in the United States, where the majority of people are on private health insurance (Chan et al., 2017). In the United States, the average cost of hearing aids exceeds $4700, which can be prohibitive for many potential users (Wilson et al., 2017). The prohibitive cost is reflected in a lower hearing aid uptake among older adults from minority ethnic groups and those in a lower SEP (Nieman & Lin, 2017; Nieman et al., 2016).

However, in addition to costs, other factors, such as a low level of education and disability, also contribute to the lower uptake of hearing aids among lower socioeconomic groups (Reichard et al., 2017). The existence of these factors explains why hearing aid use is also low in countries where most people are covered by public insurance and the cost is therefore not a barrier to hearing aid uptake (Barton et al., 2001). For instance, cost is unlikely to be a significant barrier in the United Kingdom, where the majority of hearing aids are provided in a universal healthcare setting and are free at the point of delivery. Indeed, although treatment and hearing aid provision is financially supported in the United Kingdom through the National Health Service, people in the lower socioeconomic groups use specialist health services less frequently than those in higher groups (Scholes et al., 2018). Recent evidence from the United Kingdom shows that specific demographic groups are unlikely to obtain hearing aids, proving that people of low SEP face other nonfinancial barriers. These differences do not only reflect the differences in the health systems and hearing aid provisions among countries, as suggested by Sawyer et al. (2020), but also emphasize individuals’ inability to identify their hearing difficulties as a barrier in their help-seeking process, even in countries where the hearing aids are available free of charge (Tsimpida et al., 2020a).

An explanation to the aforementioned paradox is that the perception of hearing ability acts as a strong predictor of hearing aid acquisition, even when financial factors are mitigated (Bainbridge & Ramachandran, 2014). Ng and Loke (2015) report that individuals’ SEP plays a significant role in their readiness to adopt hearing aid; the SEP may influence the self-perceived hearing problems and even the perceived benefit from the hearing aids usage. Low awareness, denial of hearing loss, self-image implications, discrimination based on age, gender, race, or disability, and acceptance of hearing loss as a normal part of the aging process impact individuals’ decision to seek hearing care (Nieman et al., 2016). Therefore, the negative attitude toward deafness and aging may play a crucial part in perpetuating individuals’ neglect of the disorder, its consequences, and possibly the onset of the related comorbidity (Fischer et al., 2011). The earlier nonaudiological determinants can be crucial for the process of change that occurs when individuals decide to seek help before further deterioration of their hearing (Feeny et al., 2012).

### Theme 4 (T4): Hearing Loss Risks the Quality and Safety of Individuals’ Health and Poses Significant Communication Barriers in Healthcare Settings, Which May Delay the Detection and Increase the Risk and Impact of Other Long-Term Conditions

Historically, hearing loss has primarily been conceptualized as impairment within a biomedical model and managed clinically within an isolated care model, with little consideration of comorbidities (Davis et al., 2016). However, hearing loss is commonly comorbid with cardiovascular disease (Bishop, 2012; Genther et al., 2013), dementia (Davies et al., 2017), depression (Armstrong et al., 2016), diabetes (Horikawa et al., 2013), falls (Lin & Ferrucci, 2012), and chronic kidney disease (Nieman & Lin, 2017). Moreover, hearing loss poses significant communication barriers in healthcare settings, and people who suffer from hearing loss are often less satisfied with their access to and the quality of healthcare provision (Barnett et al., 2014; Tsimpida et al., 2019a). The communication barrier could multiply health disparities in comorbid health conditions, as the sum of multiple health conditions, which is increasingly prevalent with advancing age, has serious consequences (Davis et al., 2016; von Gablenz et al., 2017). In a previous study involving older adults of several sociodemographic groups in Australia, people who had four or more chronic conditions reported more difficulties in navigating the healthcare system and having sufficient information for health, which are two of the nine domains of the Health Literacy Questionnaire (Beauchamp et al., 2015). Moreover, multimorbidity—which occurs mainly when an individual has poor mental health—is associated with a twofold increased risk for patient safety incidents and low quality of patient care (Panagioti et al., 2015).

People with hearing loss also face significant challenges in their communication with healthcare professionals (Barnett et al., 2014). The communication problems are also challenging for the health providers, as they may not obtain sufficient information for an accurate diagnosis (McKee et al., 2018). This issue can be highly problematic in cases of comorbidity, as it is very likely to lead to misunderstandings about diagnosis and treatment methods, or in cases of inference of patient problems which do not exist, which could lead to unnecessary testing and ineffective treatment (Barnett et al., 2014). In comorbidity, methods are needed to help providers ensure that older patients with hearing loss who are diagnosed with certain conditions do not miss important information and recommendations due to communication barriers (McKee et al., 2018). Therefore, health professionals must tailor the provision of health care to the needs of people with hearing loss (Lee & Heo, 2020).

Poor communication between providers and patients can result in a variety of adverse outcomes. It affects patients’ awareness of healthy behaviors, appropriate use of health services, understanding the importance of specific management and treatment approaches and the effective transfer of health knowledge (McKee et al., 2018). It may also result in poor adherence to treatment recommendations or have detrimental effects on patients’ clinical outcomes (McKee et al., 2018). It can thus be suggested that hearing loss negatively affects the quality and safety of health care an individual receives. The earlier communication barriers in healthcare settings may delay the detection and increase the risk and impact of other long-term conditions, which are commonly comorbid with hearing loss. Therefore, the improvement of the hearing health of the population could also improve the healthcare quality and safety for older people, as well as the broader measures of their physical, mental, and social well-being. Thus, the growing awareness of novel approaches for fostering hearing loss self-management and the emerging eHealth and mHealth applications aimed at improving hearing-related knowledge of management and treatment is very promising (Ferguson et al., 2019; Maidment et al., 2020).

### The Conceptual Model for Hearing Health Inequalities

**Figure 4.**
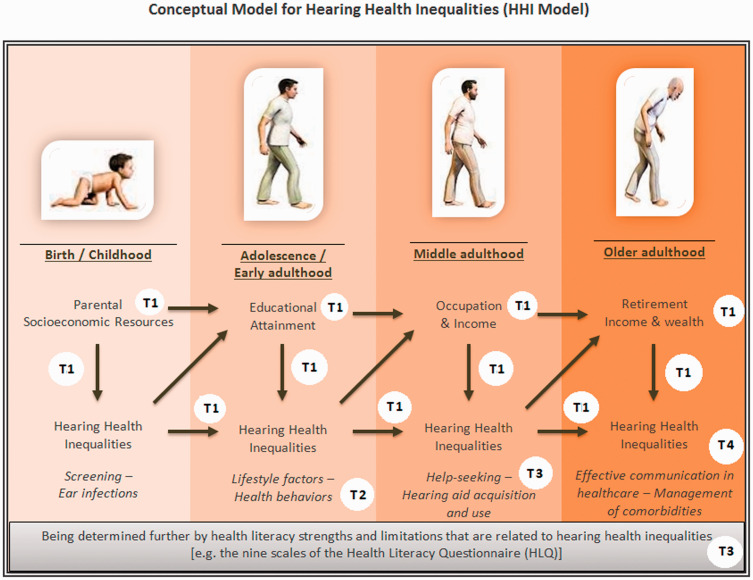
Conceptual Model for Hearing Health Inequalities (HHI Model). This work by Dialechti Tsimpida is licensed under a Creative Commons Attribution 4.0 International License. T1: Low socioeconomic position (SEP) and hearing loss form a vicious cycle, as hearing loss may be both a consequence of and a causal contributor to socioeconomic disparity. T2: Indicators of lower SEP are associated with a less healthy lifestyle, which is harmful to hearing ability. T3: Improving health literacy can mitigate hearing health inequalities and play a significant role in the adoption of beneficial hearing health behaviors, including help-seeking for hearing problems, hearing aid acquisition, and usage. T4: Hearing loss risks the quality and safety of individuals’ health and poses significant communication barriers in healthcare settings, delaying the detection and increasing the risk and impact of other long-term conditions. HLQ = Health Literacy Questionnaire.

The findings of various studies show that there are many complex factors that interact and contribute to hearing health inequalities. A conceptual model can depict the complex interaction between the socioeconomic indicators and hearing health throughout an individual’s life span, showing how these indicators impact multiple factors. The proposed model for hearing health inequalities (Figure 4) draws overtly on Åberg’s model presented in Figure 2 (Åberg Yngwe, 2004) and the concept of the dynamic relationship between health and SEP (Adler & Stewart, 2007) to provide a visual representation of the inequalities in hearing health and their evolution over time. Like other models, the Conceptual Model for Hearing Health Inequalities (HHI model) focuses on the individual’s perspective, that is, on one’s education, employment, and income (Diderichsen et al., 2012). Wealth was selected as an indicator of SEP in older adulthood (Galobardes et al., 2006). The individual experience in the HHI model is the result of several macrolevel factors, which are considered the *fundamental causes* and the *wider environmental influences* (Figure 1) which, through the multiple pathways depicted in the HHI model, lead to hearing health inequalities.

The model builds upon the four previously presented themes (Themes 1–4) that emerged from the CIS (Dixon-Woods et al., 2006) and incorporates the theoretical frameworks which have been used to explain inequalities in health. The materialist theory on which the Aberg’s model is based is also used in the HHI model, which has been stretched to life stages and supplemented by the following nonmaterialist approaches: the life-course (regarding processes with accumulative risk throughout one’s life span); the cultural-behavioral (the adoption of healthy or risk behaviors developed from cultural influences); and the psychosocial approach (in terms of the varying social positions that one may have throughout their life-course). The psychosocial approach suggests that those in a lower SEP will suffer more from health-related issues due to the psychosocial injuries derived from inequality structures, including elevated stress responses by those who work in occupations with high noise exposure and economic strains (Adler & Newman, 2002; Bartley & Blane, 2008; Elstad, 1998; Sundmacher et al., 2011). Given this, the HHI model provides a multidimensional four-component approach to social stratification, which reflects the interplay among education, occupation, income, and wealth, throughout one’s lifetime. The particular way the four themes of the CIS apply to the HHI model is described in T1, T2, T3, and T4, respectively.

#### Theme 1 (T1)

The HHI model proposes that children born to parents from lower SEP tend to experience more health-related issues. The antibiotics used to treat a bacterial infection, especially in sick babies with a genetic predisposition, may affect their hearing health. Thus, many individuals with disabling hearing impairment are disadvantaged children who have been exposed to several risk factors during their prenatal, perinatal, or neonatal period of development or have experienced inequalities in access to screening tests (Mallmann et al., 2020). Parent SEP’s role is crucial, as many of these factors are closely linked to socially and economically deprived households and neighborhoods, for example, the cytomegalovirus infection, or nutritional deficiencies (Olusanya et al., 2014).

The exact mechanisms behind the link between parental SEP and hearing health inequality are still unclear. Previous analyses that study this connection (Ecob et al., 2008; Power et al., 2007) suggest the importance of people’s social class of origin—in terms of their father’s occupation—in their hearing thresholds in adulthood but do not reach a definite conclusion regarding the possible mechanisms which cause such a link. In Ecob et al.’s (2008) study, the adjustment for noise exposure and smoking and drinking behaviors was found to reduce parental SEP’s effect on the likelihood of adulthood hearing loss by around one-third in all examined frequencies. The earlier notion led the authors to conclude that many other risk factors also need to be examined to explain the relationship between hearing loss and parental SEP.

#### Theme 2 (T2)

The consequences of hearing loss in childhood may include impairment in language skills and lower educational achievement compared with children with normal hearing (Chorozoglou et al., 2018). Having a lower educational level is a predictor of educational and social inequality in later life, as it limits one’s employment opportunities, relegating them to poorly paid jobs in their early adulthood. Manual jobs tend to be those with higher levels of noise exposure that is harmful to hearing ability, along with a possible faster deterioration in one’s overall physical health (Chandola et al., 2007). Gender differences in occupational noise exposure may explain why hearing loss is consistently cited as more prevalent among males. Furthermore, a recent study shows that living in noisy neighborhoods and being in a low SEP further enhances one’s likelihood of suffering from hearing loss (Dale et al., 2015).

#### Theme 3 (T3)

Having a lower educational status is also related to lower health literacy (Van den Broucke, 2014), which helps explain the differences between socioeconomic groups in terms of their health status (Beauchamp et al., 2015; Howard et al., 2006). Therefore, health literacy limitations may explain why individuals of a lower SEP tend to adopt an unhealthy lifestyle, with higher levels of smoking and alcohol consumption, a higher BMI and lower levels of physical activity, which all contribute to hearing loss (Howard et al., 2006; Tsimpida et al., 2019b). Occupation and income may also affect one’s access to hearing health services and hearing aids (Fischer et al., 2011). Financial barriers (direct/indirect) and one’s ability to self-diagnose may influence their motivation to seek help for hearing difficulties.

#### Theme 4 (T4)

Hearing health inequalities in middle adulthood can then affect older adults’ retirement status and income by impacting their ability to continue working or to advance occupationally (Chou et al., 2015). Lastly, hearing loss can add a further burden of disability on the lower socioeconomic groups (Marmot, 2020) by affecting not only their body functions and structures (e.g., deterioration of the ear) but also their ability to participate in society (International Classification of Diseases, 11th Revision, 2018), increasing the barriers to their use of and access to health services. This can severely affect the management of health conditions comorbid with hearing loss (Tsimpida et al., 2018a; Young, 2014). People of lower SEP may, therefore, face a double burden: first, increased levels of health impairments and, second, a lower quality of life after their health impairment occurrence (Raggi et al., 2016).

Also, hearing health inequalities may accumulate: The higher a person’s socioeconomic status, the better their hearing health can be throughout their life span. On the other hand, those who are persistently exposed to inadequate socioeconomic resources during their childhood and adulthood face a disproportionately higher chance of suffering from hearing loss. It is now clear how the low SEP and hearing loss form a vicious cycle, as hearing loss can be a consequence and a causal contributor to socioeconomic disparity.

Health literacy has been defined by the WHO as *the cognitive and social skills that determine individuals' motivation and ability to gain access to, understand and use information in ways that promote and maintain good health* (Nutbeam, 1998). The gray text box about health literacy highlights—from individual’s perspective—that health literacy skills act as modifiers, underpinning the relationship between socioeconomic inequalities and hearing health over time (Arcaya et al., 2015). Given this, the conceptual model for hearing health inequalities is not a “fixed procrustean framework” that enforces uniformity in explaining hearing health inequalities; instead, it recognizes the multifactorial interindividual variance in hearing health inequalities (Diez Roux, 2012). According to the HHI model to reduce SEP’s impact on hearing health, healthy hearing should be promoted as a lifelong process.

## Discussion and Implications

This study suggests that (a) a vicious cycle between hearing loss and socioeconomic inequalities and lifestyle factors exists, (b) SEP prompts healthy or unhealthy lifestyles which affect people’s hearing ability, (c) people with hearing loss are more at risk of receiving low quality and less safe health care, and (d) increasing health literacy could improve the diagnosis and prognosis of hearing loss and prevent the adverse consequences of hearing loss on people’s health. The HHI model identifies determinants of hearing loss using a life-course approach, which aims to shine new light on the current hearing health research debates. This model can be used as a tool for preventing, identifying, and managing hearing health inequalities and for policy formulation to reduce hearing loss risks.

### Limitations

There are significant limitations in terms of the type and quality of existing published literature on the topic. The field of hearing health inequalities is an emerging research field, and most of the evidence cited in the article stems from cross-sectional studies which demonstrate associations. However, although the CIS approach demands attention to study design, it also allows for the inclusion of less methodologically robust papers as long as they are essential in their theoretical contribution (Dixon-Woods et al., 2006). Thus, in line with the CIS principles, this article views the task of critical synthesis not as aggregation, but as induction and interpretation, aiming to integrate the concepts and provide new insights and unified ways of understanding the amorphous and complex phenomenon of hearing health inequalities, rather than simplifying it.

The line of argument emerged from the synthesis of the existing evidence into a conceptual form. During this process, the researchers’ subjectivity was intimately involved and reflexively accounted for, which may be controversial (Depraetere et al., 2020). However, the CIS approach explicitly acknowledges the *authorial voice* in examining a network of synthetic constructs (themes) and the relationships between them and places a great deal of emphasis on the researcher’s interpretation (Barnett-Page & Thomas, 2009). Besides, it is common for conceptual models to emphasize some factors than others, which sets the bounds for a complex research topic and specifies which relationships will be espoused as fundamental (Diez Roux, 2012). In his attempt to offer a useful account of the literature, George Box, one of the great statistical minds of the 20th century, once stated that “all models are wrong, but some are useful” (Wasserstein, 2010).

The HHI model will hopefully guide future research to examine the directionality of associations and conduct longitudinal studies and intervention trials to explore further many of the assertions shared in this article, or potential differences due to residence (Brennan-Jones et al., 2016). It may also be helpful for future epidemiological research to differentiate the hearing loss based on the age of onset and etiology. The vast majority of the global population (80%) lives in low- and middle-income countries (Wilson et al., 2017), lacking resources for diagnosing and treating hearing loss and experiencing huge hearing health inequalities. It would therefore be useful for robust evidence to be obtained on populations living in low- and middle-income countries.

Hopefully, the HHI model will prompt researchers to develop new questions which need to be answered or stimulate them to think in new ways about the existing questions (Diez Roux, 2012). Future research will be then better placed to produce aggregative syntheses using conventional systematic review methods and techniques such as meta-analysis (Dixon-Woods et al., 2006), adding even more elements to the HHI model.

### Implications for Health Policy

Viewing hearing health care as a health behavior provides novel insight into the development of effective interventions to increase individuals’ help-seeking behavior, which will allow them to reduce and prevent the adverse effects of hearing loss.

Interventions designed to reduce hearing health inequalities can be implemented across three levels, which, following Geronimus’ distinction (Geronimus, 2000), are (a) mitigation, (b) preventing, and (c) undoing inequalities (see Figure 1). *Mitigation* refers to actions which aim to reduce the impact of social inequalities on people’s hearing health and social outcomes by recognizing these barriers. For example, general practice appointments would be more effective if the service provider were aware that the patient cannot read well or is not fully conversant with the language to seek help for hearing difficulties. Thus, health service interventions, which aim to increase awareness among health professionals about the high prevalence of hearing loss and the insufficient management of hearing difficulties among different social groups, are needed (Tsimpida et al., 2019b).

Second, *preventing* involves acknowledging those who have limited access to hearing health aid and whose working and living conditions put them more at risk of suffering from poor hearing health. Several primary prevention activities—such as improved prenatal, perinatal, or neonatal care; universal vaccination programs; and antibiotic stewardship practices—can be implemented to reduce the incidence of hearing loss from preconception to adulthood. In addition, secondary and tertiary prevention activities—such as prompt intervention, fitting of hearing devices (hearing aids, cochlear implants, etc.), and training in sign language and special or inclusive education—are needed and should be actively encouraged (Olusanya et al., 2014).

Finally, *undoing* hearing health inequalities refers to the fact that there could be a differential economic policy that aims to decrease the wealth gap, thereby reducing the hearing health gap. Therefore, governmental policies aimed at reducing socioeconomic and education inequalities are needed to improve the most vulnerable groups’ hearing health. Such policies can make essential contributions to preventing further increases in hearing health inequalities (Lorenc et al., 2013). Otherwise, any action that does not focus on the social determinants but only on hearing health improvement may further increase the existing hearing health inequalities of the population’s hard-to-reach subgroups (Lorenc et al., 2013).

### Implications for Societies

Because the burden of high levels of hearing loss affects the economic growth and development of a country, tackling hearing health inequalities has important implications for individuals and society as a whole. These negative impacts arise from the interaction of hearing loss with the broader social environment and can be significantly mitigated through the early identification and the appropriate management of hearing problems (WHO, 2013). Hearing loss generates costs to society, such as higher welfare payments, healthcare expenditures, and lost tax revenues. Characteristically, it is estimated that unaddressed hearing loss costs the global economy 750 billion annually (Ramsey et al., 2018). If this burden of hearing loss persists, it could slow economic growth, with developing countries suffering the most (Ramsey et al., 2018).

## Conclusion

The increase in the aging population and the burden of hearing loss and the concentration of ill-health among older adults have highlighted the urgent need to investigate factors that contribute to socioeconomic inequalities in hearing health. Although previous studies have found correlations between (a) socioeconomic inequalities and hearing loss, (b) hearing loss and comorbidity, and (c) hearing impairment and related socioeconomic disparities, this review is the first to examine the mechanisms and explain the relationship between socioeconomic inequalities and hearing health in a life-course perspective, synthesizing the existing evidence.

Apart from the physiological and pathological aging of sense organs, the HHI model provides a visual representation of several modifiable determinants of hearing loss in distinct life stages, supporting the argument that a substantial proportion of hearing loss in older adulthood is preventable, treatable, and even postponable. Understanding that hearing deterioration occurs over a prolonged period of time is an essential step in addressing the burden of hearing loss not within an isolated model of care which focuses on the acquired hearing loss among older adults, but as a lifelong process.

Although reducing hearing health inequalities is a complex ambition, the life-course approach can lead to the development of appropriate interventions and public health strategies that can have significant health policy and practice implications. The management of hearing loss must involve integrated care, which entails considering an individual’s entire health profile and providing ongoing support for each person’s adaptation and self-management. In that way, we will ensure that a more substantial proportion of the population receives high-quality health care and maximizes the opportunity for healthy aging.

## Supplemental Material

sj-pdf-1-tia-10.1177_23312165211002963 - Supplemental material for Conceptual Model of Hearing Health Inequalities (HHI Model): A Critical Interpretive Synthesis Click here for additional data file. Supplemental material, sj-pdf-1-tia-10.1177_23312165211002963 for Conceptual Model of Hearing Health Inequalities (HHI Model): A Critical Interpretive Synthesis by Dialechti Tsimpida, Evangelos Kontopantelis, Darren M. Ashcroft and Maria Panagioti in Trends in Hearing
