# Supplementary material for: Conceptual Model of Hearing Health Inequalities (HHI Model): A Critical Interpretive Synthesis
Source: Trends Hear. 2021 May 28;25:23312165211002963. doi: 10.1177/23312165211002963 (PMC8165532; doi:10.1177/23312165211002963)
Supplement: sj-pdf-1-tia-10.1177_23312165211002963 - Supplemental material for Conceptual Model of Hearing Health Inequalities (HHI Model): A Critical Interpretive Synthesis [file sj-pdf-1-tia-10.1177_23312165211002963.pdf]

SUPPLEMENTARY MATERIAL

Supplementary Table 1 Key points derived from the 53 studies that support the four research themes of the translational analysis

No	Author (s), Year	Country	Key point made by authors	Theme 1	Theme 2	Theme 3	Theme 4
1	**Andrade & Lopez-Ortega, 2017**	Brazil and Mexico	Association of lower educational attainment with higher odds of hearing loss	√			
2	**Armstrong, et al., 2016**	United States	Comorbidity of hearing loss with depression				√
3	**Bainbridge & Ramachandran, 2014**	United States	Role of income in the lower hearing aid acquisition and usage and disparities among people with hearing loss in access to hearing health services	√		√	
4	**Barnett et al., 2014**	United States	Barriers in access to health services and lower satisfaction among those with hearing loss with the quality of health care provision			√	√

No	Author (s), Year	Country	Key point made by authors	Theme 1	Theme 2	Theme 3	Theme 4
5	**Barton et al., 2001**	Denmark Finland Norway Sweden United Kingdom	Factors for the lower hearing aid use			√	
6	**Benova et al., 2015**	United Kingdom	Role of socioeconomic position in the health-seeking process among older adults with hearing loss			√	
7	**Bishop, 2012**	United States	Comorbidity of hearing loss with cardiovascular disease				√
8	**Chan et al., 2017**	United States	Financial barriers to hearing healthcare and factors affecting the help-seeking behaviour	√		√	
9	**Chien & Lin, 2012**	United States	Barriers in access to health services after the onset of hearing loss			√	
10	**Chorozoglou et al., 2018**	United Kingdom	Consequences of hearing loss in language skills of children and impact on their educational achievement	√			

No	Author (s), Year	Country	Key point made by authors	Theme 1	Theme 2	Theme 3	Theme 4
11	**Chou et al., 2015**	United States	Correlation between social indicators and the likelihood of developing hearing impairment, the association of hearing loss with significant adverse outcomes	√		√	
12	**Cruickshanks et al., 1998**	United States	The relation between occupational exposure to noise and an increased likelihood of having hearing loss	√			
13	**Curhan et al., 2013**	United States	Role of exercise in the likelihood of hearing loss		√		
14	**Davies et al., 2017**	United Kingdom	Comorbidity of hearing loss with dementia				√
15	**Davis et al., 2016**	Review study	Role of nutrition and exercise in the onset of hearing loss, consequences of hearing loss in the decision about early retirement and subsequently the income of older adults, communication barriers in comorbidities	√	√		√

No	Author (s), Year	Country	Key point made by authors	Theme 1	Theme 2	Theme 3	Theme 4
16	**Eisele et al., 2015**	Germany	Role of hearing loss in good health maintenance and social participation	√			√
17	**Emmett & Francis, 2015**	United States	Role of hearing loss in the ability of the individuals to continue working or to advance occupationally	√			
18	**Feeny et al., 2012**	Canada	Disability in hearing is associated with mortality				√
19	**Fischer et al., 2011**	United States	Role of negative attitudes towards deafness and ageing			√	
20	**Genther et al., 2013**	United States	Comorbidity of hearing loss with cardiovascular disease				√
21	**Gopinath et al., 2010**	Australia	Smoking and association with hearing loss		√		

No	**Author (s), Year**	**Country**	**Key point made by authors**	**Theme 1**	**Theme 2**	**Theme 3**	**Theme 4**
22	**Harrison et al., 2020**	Malawi	Barriers in access to health care for people with hearing loss			√	
23	**Helvik et al., 2009**	Norway	Occupational exposure to noise and increased likelihood of hearing loss	√			
24	**Horikawa et al., 2013**	Meta-analysis	Comorbidity of hearing loss with diabetes				√
25	**Kupriianova et al., 2013**	Russia	Social indicators and relation with hearing loss	√			√
26	**Lee et al., 2020**	South Korea	Increased health needs among those with hearing loss, using the International Classification of Functioning, Disability and Health				√
27	**Lin & Ferrucci, 2012**	United States	Association of hearing loss with falls in the elderly				√

No	Author (s), Year	Country	Key point made by authors	Theme 1	Theme 2	Theme 3	Theme 4
28	**Luo et al., 2020**	China	Socioeconomic inequalities in hearing loss among working-aged adults			√	
29	**Mallmann et al., 2020**	Brazil	Socioeconomic inequalities in access to neonatal screening tests	√			
30	**Mamo et al., 2016**	United States	Higher prevalence of untreated hearing loss among low-income older adults	√			
31	**Martin et al., 2012**	United Kingdom	Association of higher educational attainment with lower odds of hearing impairment in adults of working age	√			
32	**McKee et al., 2018**	United States	Disease burden and low health-related quality of life among older adults with hearing loss	√			√
33	**Ng & Loke, 2015**	Review study	Role of socioeconomic position in an individual's readiness to hearing aid adoption and usage			√	

No	Author (s), Year	Country	Key point made by authors	Theme 1	Theme 2	Theme 3	Theme 4
34	**Nieman & Lin, 2017**	Review study	Disparities in access to health services and comorbidity of hearing loss with chronic kidney disease			√	√
35	**Nieman et al., 2016**	United States	Lower hearing aid uptake among minority older adults and those in a lower SEP			√	
36	**Pichetti et al., 2016**	France	hearing loss is a major barrier in access to care among those in a low socioeconomic position			√	
37	**Pierre et al., 2012**	Sweden	Association of low education attainment with occupation involving high levels of noise exposure	√			
38	**Raggi et al., 2016**	Finland<br>Poland<br>Spain	Lower quality of life after a health impairment occurrence among those with low socioeconomic position	√			
39	**Reichard et al., 2017**	United States	Lower uptake of hearing aids among lower socioeconomic groups			√	

No	Author (s), Year	Country	Key point made by authors	Theme 1	Theme 2	Theme 3	Theme 4
40	**Rosenhall et al., 1999**	Sweden	Hearing difficulties among workers of manual occupations	√			
41	**Scholes et al., 2018**	United Kingdom	Association of socioeconomic position with hearing loss and the differences in the use of specialist health services	√		√	
42	**Shaw et al., 2013**	Canada	Challenges among those with hearing loss in work performance and productivity in the workplace	√			
43	**Smith et al., 2016**	United Kingdom	Consequences of hearing loss in the income of older adults	√			
44	**Spankovich & Le Prell, 2013**	United States	Impact of physical activity on hearing loss		√		
45	**Sung et al., 2013**	Korea	The damaging effect of occupational noise-exposure in hearing		√		

No	Author (s), Year	Country	Key point made by authors	Theme 1	Theme 2	Theme 3	Theme 4
46	**Tsimpida et al., 2018a**	Greece	Determinants of Health-related Quality of Life (HRQoL) among Deaf and Hard of Hearing Adults	√	√		
47	**Tsimpida et al., 2018b**	Greece	Barriers to the use of health services among adults with hearing loss			√	√
48	**Tsimpida et al., 2019a**	Greece	Inequalities in access to health services faced by the population with hearing loss			√	√
49	**Tsimpida et al., 2019b**	United Kingdom	Socioeconomic and lifestyle factors associated with hearing loss in older adults	√	√		
50	**Üçler et al., 2016**	Turkey	Association with an unhealthy diet with hearing loss		√		
51	**von Gablenz & Holube, 2017**	Germany	The higher prevalence of untreated hearing loss among adults in a low socioeconomic position and possible associations with more severe health problems	√			√

No	Author (s), Year	Country	Key point made by authors	Theme 1	Theme 2	Theme 3	Theme 4
52	**Wilson et al., 2017**	Review study	Role of cost in the lower hearing aid uptake among minority older adults and those in a lower socioeconomic position			√	
53	**Zhan et al., 2011**	United States	Role of lower education with higher odds of hearing loss and association of alcohol consumption with hearing loss	√	√		

Supplementary Table 2 Summary of the quality appraisal of included studies *

No	Author (s), Year	Selection bias [a]	Design [b]	Covariates [c]	Data collection methods [d]	Total quality score
1	**Andrade & Lopez-Ortega, 2017**	**1**	**0**	**1**	**0**	**2**
2	**Armstrong, et al., 2016**	**0**	**1**	**1**	**0**	**2**
3	**Bainbridge & Ramachandran, 2014**	**1**	**0**	**1**	**1**	**3**
4	**Barnett et al., 2014**	**1**	**0**	**1**	**1**	**3**
5	**Barton et al., 2001**	**1**	**1**	**0**	**1**	**3**
6	**Benova et al., 2015**	**1**	**0**	**1**	**0**	**2**
7	**Bishop, 2012**	N/A	N/A	N/A	N/A	N/A
8	**Chan et al., 2017**	**0**	**0**	**0**	**0**	**0**

No	Author (s), Year	Selection bias [a]	Design [b]	Covariates [c]	Data collection methods [d]	Total quality score
9	**Chien & Lin, 2012**	**1**	**1**	**0**	**1**	**3**
10	**Chorozoglou et al., 2018**	**0**	**1**	**1**	**1**	**3**
11	**Chou et al., 2015**	**1**	**1**	**1**	**0**	**3**
12	**Cruickshanks et al., 1998**	**1**	**1**	**1**	**1**	**4**
13	**Curhan et al., 2013**	**1**	**1**	**1**	**0**	**3**
14	**Davies et al., 2017**	**1**	**1**	**1**	**1**	**4**
15	**Davis et al., 2016**	N/A	N/A	N/A	N/A	N/A
16	**Eisele et al., 2015**	**1**	**1**	**1**	**0**	**3**
17	**Emmett & Francis, 2015**	**1**	**0**	**1**	**1**	**3**

No	Author (s), Year	Selection bias [a]	Design [b]	Covariates [c]	Data collection methods [d]	Total quality score
18	**Feeny et al., 2012**	**1**	**1**	**1**	**0**	**3**
19	**Fischer et al., 2011**	**1**	**1**	**1**	**1**	**4**
20	**Genther et al., 2013**	**1**	**1**	**1**	**1**	**4**
21	**Gopinath et al., 2010**	**1**	**1**	**1**	**1**	**4**
22	**Harrison et al., 2020**	**0**	**0**	**0**	**0**	**0**
23	**Helvik et al., 2009**	**1**	**1**	**0**	**1**	**3**
24	**Horikawa et al., 2013**	N/A	N/A	N/A	N/A	N/A
25	**Kupriianova et al., 2013**	**0**	**0**	**0**	**1**	**1**
26	**Lee et al., 2020**	**0**	**0**	**0**	**0**	**0**

No	Author (s), Year	Selection bias [a]	Design [b]	Covariates [c]	Data collection methods [d]	Total quality score
27	**Lin & Ferrucci, 2012**	**1**	**0**	**1**	**1**	**3**
28	**Luo et al., 2020**	**1**	**0**	**1**	**1**	**3**
29	**Mallmann et al., 2020**	**1**	**0**	**1**	**1**	**3**
30	**Mamo et al., 2016**	**1**	**1**	**0**	**1**	**3**
31	**Martin et al., 2012**	**1**	**1**	**1**	**0**	**3**
32	**McKee et al., 2018**	**1**	**1**	**1**	**0**	**3**
33	**Ng & Loke, 2015**	N/A	N/A	N/A	N/A	N/A
34	**Nieman & Lin, 2017**	N/A	N/A	N/A	N/A	N/A
35	**Nieman et al., 2016**	**1**	**1**	**0**	**1**	**3**

No	Author (s), Year	Selection bias [a]	Design [b]	Covariates [c]	Data collection methods [d]	Total quality score
36	**Pichetti et al., 2016**	**1**	**0**	**1**	**0**	**2**
37	**Pierre et al., 2012**	**1**	**0**	**1**	**0**	**2**
38	**Raggi et al., 2016**	**1**	**0**	**0**	**0**	**1**
39	**Reichard et al., 2017**	**1**	**1**	**1**	**0**	**3**
40	**Rosenhall et al., 1999**	**1**	**1**	**0**	**0**	**2**
41	**Scholes et al., 2018**	**1**	**0**	**1**	**1**	**3**
42	**Shaw et al., 2013**	**0**	**0**	**0**	**0**	**0**
43	**Smith et al., 2016**	**0**	**0**	**0**	**0**	**0**
44	**Spankovich & Le Prell, 2013**	**1**	**0**	**1**	**1**	**3**

No	Author (s), Year	Selection bias [a]	Design [b]	Covariates [c]	Data collection methods [d]	Total quality score
45	**Sung et al., 2013**	**0**	**0**	**1**	**1**	**2**
46	**Tsimpida et al., 2018a**	**0**	**0**	**1**	**0**	**1**
47	**Tsimpida et al., 2018b**	**0**	**0**	**1**	**0**	**1**
48	**Tsimpida et al., 2019a**	**0**	**0**	**1**	**0**	**1**
49	**Tsimpida et al., 2019b**	**1**	**0**	**1**	**1**	**3**
50	**Üçler et al., 2016**	**0**	**1**	**0**	**1**	**2**
51	**von Gablenz & Holube, 2017**	**0**	**0**	**1**	**1**	**2**
52	**Wilson et al., 2017**	N/A	N/A	N/A	N/A	N/A
53	**Zhan et al., 2011**	**1**	**1**	**1**	**1**	**4**

* Each point given indicates the presence of the relevant criterion

[a] Selection bias: likely to be representative of the target population and have a response rate or data capture among eligible participants of 70% or greater.

[b] Design: cohort analytic, case-control, cohort, or an interrupted time series.

[c] Covariates: control for a minimum of 3 critical covariates in the analysis, including sociodemographic characteristics (e.g. age, sex, education).

[d] Data collection methods: psychoacoustic hearing assessment tools, which are valid and reliable, or data from medical records.
